# Spine and Sacroiliac Joints Lesions on Magnetic Resonance Imaging in Early Axial-Spondyloarthritis During 24-Months Follow-Up (Italian Arm of SPACE Study)

**DOI:** 10.3389/fimmu.2020.00936

**Published:** 2020-05-15

**Authors:** Mariagrazia Lorenzin, Augusta Ortolan, Mara Felicetti, Stefania Vio, Marta Favero, Pamela Polito, Carmelo Lacognata, Vanna Scapin, Andrea Doria, Roberta Ramonda

**Affiliations:** ^1^Rheumatology Unit, Department of Medicine–DIMED, University of Padova, Padova, Italy; ^2^Radiology Unit, University of Padova, Padova, Italy

**Keywords:** diagnostic imaging, inflammatory biomarkers, low back pain, spine, disease process

## Abstract

**Objectives:** Our study aimed to identify: (1) the prevalence of spine and pelvis magnetic resonance imaging (MRI-spine and MRI-SIJ) inflammatory and structural lesions in patients (pts) with a diagnosis of axial spondyloarthritis (axSpA); (2) the predictive factors for a severe disease pattern with a higher probability of radiographic progression.

**Materials and Methods:** Seventy-five pts with low back pain (LBP) (≥3 months, ≤2 years, onset ≤45 years) underwent physical examination, questionnaires, laboratory tests, X-rays, MRI-spine, and MRI-SIJ at baseline (T0) and during a 24-months follow-up. Two expert rheumatologists made axSpA diagnosis and classification (according ASAS criteria). MRI-spine, MRI-SIJ and X-rays were scored independently by 2 readers following the SPARCC, mSASSS, and mNY-criteria. According to ASAS criteria, 21 pts fulfilled imaging arm only and 29 clinical arm with/without imaging arm; 25 pts did not fulfill ASAS criteria.

**Results:** At T0 the mean ± SD LBP onset was 28.51 ± 8.05 years, 45.3% pts were male, 38.7% were HLA-B27+; 56% showed bone marrow oedema (BMO) at MRI-spine and 64% at MRI-SIJ. Signs of enthesitis were found in 58% pts in the thoracic spine. Eighteen (24%) pts presented BMO at MRI-spine with a negative MRI-SIJ. The prevalence of BMO lesions and the SPARCC SIJ and spine score decreased during the follow-up in the 2 cohorts meeting ASAS criteria. An early onset of LBP, a lower use of NSAIDs, a BASDAI>4 were identified as predictors of spine structural damage; the high SPARCC SIJ score appeared to be a predictor of SIJ structural damage. A higher mSASSS was predicted by a lower age of onset of LBP. Predictor of higher SPARCC spine was a higher NSAIDs and of higher SPARCC SIJ score the HLA-B27 positivity with increased inflammatory biomarkers.

**Conclusions:** At T0 a significant prevalence of BMO lesions was observed both in SIJ and spine, with predominant involvement of thoracic district. Since positive MRI-spine images were observed in the absence of sacroiliitis, these findings seem to be relevant in the axSpA diagnosis. Early age of disease onset, long duration of LBP, increased inflammatory biomarkers, higher use of NSAIDs, male gender, HLA-B27 positivity, SPARCC SIJ score>2 appeared predictors of radiological damage and activity.

## Introduction

**Figure 1:**
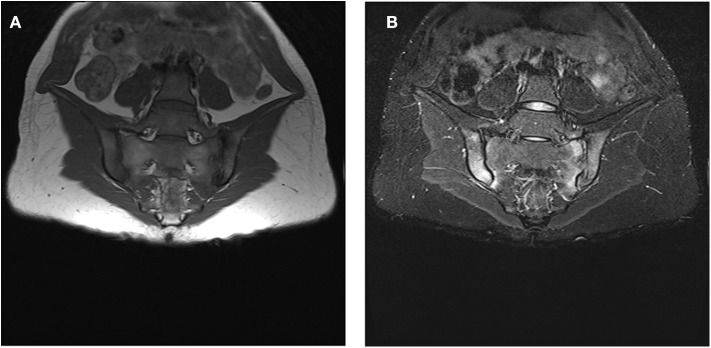
**(A,B)**: Bone marrow oedema (BMO) signs at both SIJ. **(A)** hypointense signal at the sacral and iliac superior and inferior third region of both right and left SIJ in sequence T1. **(B)** corresponding iperintense signal at the sacral and iliac superior and inferior third region of both right and left SIJ in sequence STIR. The written informed consent was obtained from the individual for the publication of these images.

**Figure 2:**
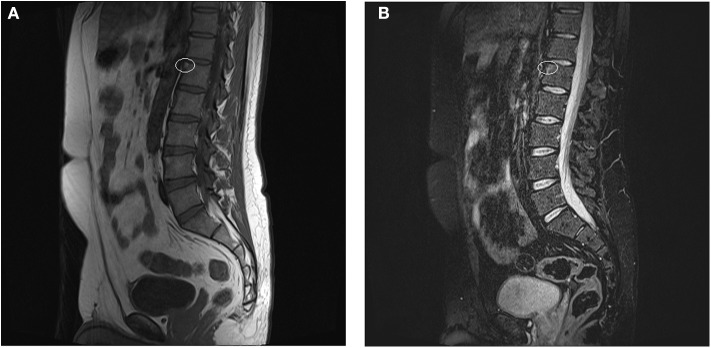
**(A,B)** Anterior spondylitis. **(A)** hypointense signal at the anterior-superior corner of L1 (white ring) in sequence T1 **(B)** corresponding hyperintense signal at the anterior-superior corner of L1 in sequence STIR (white ring). The written informed consent was obtained from the individual for the publication of these images.

Spondyloarthritis (SpA) is a group of chronic inflammatory rheumatic diseases that share overlapping features and can be subdivided into axial SpA (axSpA) and peripheral SpA (pSpA) (1–3). AxSpA mainly affects the spine and the sacroiliac joints (SIJ), has an early onset at young age and can be further subdivided between non-radiographic (nr-axSpA) and radiographic axSpA (r-axSpA), the latter also known as ankylosing spondylitis (AS) (2). With the development of new effective treatment strategies, the need to identify patients in an earlier stage of disease has increased (3, 4). Early onset nr-axSpA may present as a very active disease with axial pain symptoms responding quickly and effectively to therapy. Therefore, the identification of these early forms becomes a priority in order to establish the most appropriate treatment (1, 3). *The Assessment of SpondyloArthritis International Society* (ASAS) has established classification criteria to identify patients with early stage axSpA (3); the imaging arm of the criteria requires the presence of sacroiliitis on magnetic resonance imaging (MRI) or on X-rays in addition to one SpA feature for patients with chronic low back pain (LBP) with onset ≤ 45 years of age. Conventional X-rays of SIJ, still frequently used to detect sacroiliitis, do not appear to provide adequate information to classify patients with suspected early axSpA, as they detect only structural bone damage, indicative of a more advanced disease stage (3, 5). Thus, MRI represents an important additional screening option since it can detect inflammatory lesions of SIJ in patients with early-onset axSpA without evidence of radiographic sacroiliitis (6). Positive MRI-SIJ scans were defined by the *ASAS/Outcome Measures in Rheumatology MRI working group* (ASAS/OMERACT) as the presence of inflammatory lesions such as *bone marrow edema* (BMO) which is highly suggestive of SpA (6) (Figures 1A,B). Whether structural SIJ lesions should be added to this definition and whether structural and inflammatory spinal lesions could contribute to detecting axSpA remains a matter of debate (7). Inflammatory spinal lesions on MRIs may nevertheless occur in the absence of SIJ involvement (8, 9). These lesions include BMO adjacent to vertebral endplates at the attachment of the annulus fibrosus to the vertebral rim and at the insertion of anterior and posterior longitudinal ligaments, both within the facet joints (Figures 2A,B). Since there is evidence that spondylitis may also occur prior to -or even without- sacroiliitis, it was deemed important to define the characteristics of a MRI-spine considered positive for inflammation. The ASAS/OMERACT working group thus defined MRI-spine criteria of inflammatory lesions (spondylitis) and structural changes (fat deposition) (7). Imaging of the thoracic spine, often involved in axSpA, has instead not yet been taken into consideration in evaluation of structural damage (10–12). The goal of this study was to determine the prevalence of spine and SIJ lesions on MRI and their correlation with clinical and disease activity indices in patients with early axSpA included in the *SpondyloArthritis-Caught-Early* (SPACE) Italian cohort at baseline (T0) and during a 24-months follow-up. Secondary objectives included evaluation of: (a) the role of imaging in the diagnostic process of axSpA with or without SIJ involvement; (b) evolution of MRI features over time and their relationship to radiographic damage; (c) predictors of radiological progression and severe disease.

## Materials and Methods

### Patients

The SPACE study is an on-going observational prospective cohort multi-center study II level evidence (Netherlands, Norway, Sweden, Italy). Patients who were at least 16 years old, suffering by chronic inflammatory LBP (≥3 months, ≤2 years, onset < 45 years) of unknown origin and referred to a rheumatologist were included. In the present study only patients from our center were considered. Approval by local Medical Ethics Committee (Azienda Ospedaliera di Padova [approval no. 2438P]) was obtained. Informed consent was required from the patients prior to inclusion. Eligible patients underwent physical examinations and laboratory tests following a standardized protocol at baseline (T0) and at 6, 12, 24 months (T6, T12, T24). The patients also completed questionnaires on disease activity, physical functioning, pain and disease-related impairment. SIJ and spinal plain radiographs and MRIs were performed at T0, T12 and T24. Axial pain and MRI lesions were localized in 4 sites: cervical/thoracic/lumbar spine and SIJ. X-rays and MRI images were read by two expert radiologists (SV and VS), while 2 experienced rheumatologists (RR and ML) made axSpA diagnosis and classified subjects according to ASAS criteria (3). The patients were subdivided into three cohorts: those fulfilling only the imaging arm of ASAS axSpA criteria (*axSpA imaging arm*), those fulfilling the clinical arm of ASAS axSpA criteria in presence/absence of imaging arm (*axSpA clinical*±*imaging arm)* and those not fulfilling ASAS axSpA criteria (*not full ASAS axSpA*). A detailed description of the recruitment and clinical assessment of the SPACE cohort has previously been published (13, 14). At T0 all the patients were being treated with non-steroidal anti-inflammatory drugs (NSAIDs). Afterwards, patients were being treated according to best clinical practice, with no limitation on pharmacological treatments, physical therapies or other treatments.

### Methods

#### Physician Clinical, Questionnaires', and Biochemical Assessments

The clinical evaluation focused on an examination of the spine, SIJ and entheses, using the *Bath Ankylosing Spondylitis Metrology Index* (*BASMI)* and the *Maastricht Ankylosing enthesitis Spondilities Score (MASES*). The patients' disease activity and physical functioning were assessed using self-reported questionnaires and composite indices: the *Bath Ankylosing Spondylitis Disease Activity Index (BASDAI)*, the *Bath Ankylosing Spondylitis Functional Index (BASFI)*, the *Ankylosing Spondylitis disease activity score (ASDAS)*, the *Visual Analogue Scale (VAS) pain*, the *VAS night pain*, the *VAS disease activity*, the *Bath Ankylosing Spondylitis Patient Global Score (BASG1)*, the *BASG2*, the *Health Assessment Questionnaire (HAQ)*. Biochemical parameters included the erythrocyte sedimentation rate (ESR) (Westergren method in mm after 1 h; normal range 0–15 mm/h) and C-reactive protein (CRP) (ELISA in mg/l; Research & Diagnostic Systems, Inc., expressed in mg/L, normal range 0–6 mg/L). DNA was also collected to perform HLA-B (complement-dependent microlymphocytic assay). Bank serum was be stored at −70C. Serum for the assays was separated by centrifugation at 3,000 rpm for 10 min. All blood samples were analyzed twice using the same method.

#### MRI and Radiographs Assessments

MRI-SIJ and MRI-spine were performed using a 1.5 T scanner Magnetom Harmony, Siemens AG Medical Solutions, Munich, with phased-array surphace coil, acquiring T1-weighted turbo spin echo (T1TSE; TR 583/TE 9.4) and short-tau inversion recovery (STIR; TR 2980/TE47) sequences. The coronal oblique and sagittal views of the SIJ and spine were taken, with a slice thickness of 4 mm. Lateral view radiographs of the cervical and lumbar spine and anterior-posterior view radiographs of the pelvis were taken. The images were obtained with a Philips vertical bucky, with a focus-film distance of 140 cm, film size of 18 × 43 cm. MRI images were analyzed according to ASAS/OMERACT criteria (6, 7). The inflammatory positive MRI images were scored using the *Spondyloarthritis Research Consortium of Canada* (SPARCC) score (15, 16). For spine and SIJ X-rays the *Stoke Ankylosing Spondylitis Spinal Score (SASSS) system* modified by Creemers (mSASSS) (10) and mNY criteria (5) were used. All readers were blinded for clinical and laboratory data, and for the results of the other imaging methods. The temporal sequence of the images was unblinded to radiologists for the scoring. When two readers (SV and VS) both scored an SIJ image positive according to ASAS/OMERACT and mNY, the image was considered positive. In case of disagreement, an adjudicator was introduced (CL). If the primary readers agreed on a positive (or negative) MRI-SIJ, the mean SPARCC scores were calculated based on the scores of these primary readers. In cases of disagreement, the mean scores were based on the consensus scores of the adjudicator and 1 primary reader. A similar process was followed for calculating the mean SPARCC scores in the MRI-spine. The adjudicator (CL) was also introduced in case of disagreement between the readers regarding the presence/absence of spine radiological lesions on X-rays. In cases of agreement, the mean score between the 2 readers was calculated for mSASSS. Intra and inter-observer reliability was assessed.

### Statistical Analysis

The Cohen's Kappa test was used to assess the intra and inter-observational reliability between the two rheumatologists and radiologists. The non-parametric Kruskal-Wallis test (one-way ANOVA for ranks) for repeated measurements followed by the Dunn's multiple Comparison test were used to compare the clinical (BASMI, MASES), bioumoral (ESR,CRP), functional (BASFI, HAQ, BASG1, BASG2, VAS pain, VAS night pain, VAS disease activity) and disease activity indices (BASDAI, ASDAS) at T0, T6, T12 and T24. The same method was used to compare the imaging scores (mSASSS, score SIJ mNY, SPARCC SIJ, and SPARCC spine) at T0, T12 and T24 in all patients and among the three cohorts (*axSpA imaging arm, axSpA clinical*±*imaging arm and not full ASAS axSpA*). A linear regression analysis was performed to identify baseline predictors of inflammation and radiological progression of the disease evaluated using as outcomes mSASSS, score SIJ mNY, SPARCC SIJ and SPARCC spine indices at T24. The following independent variables were considered in the univariable analysis: female sex, age of LBP onset, duration of LBP, presence of HLA-B27, elevated inflammation indices, BASDAI>4, use of NSAIDs, SPARCC SIJ score>2. The significant independent variables (*p* < 0.1) at univariable analysis were introduced in the multivariable models. The radiographic progression of SIJ from T0 to T24 was defined as (17): (1) transition from nr-axSpA to r-axSpA according to the mNY criteria; (2) change of >1 degree of sacroiliitis but ignoring the transition from degree 0 to 1 of sacroiliitis. The radiographic progression of the spine from T0 to T24 was defined as (18) an increase in mSASSS score >2 over the course of 2 years of observation. All statistical analyses were performed using the SPSS 13.0 program (SSPS Inc, IL, USA). The values of *p* < 0.05 were considered statistically significant.

## Results

Seventy-five patients with chronic inflammatory LBP were enrolled. According to the ASAS criteria for axSpA, **21** (28%) patients were classified as *axSpA imaging arm*, **29** (38.7%) patients as *axSpA clinical*±*imaging arm* and **25** (33.3%) patients as *not full ASAS criteria for axSpA*. The agreement between the two clinicians was good (k 0.75). The average age at LBP onset was 28.51 ± 8.05 years, 45.3% were male, 38.7% were HLA-B27+. Thirty-nine (52%) of patients presented an exclusive axial involvement, while 36 (48%) of patients also had peripheral involvement. A high prevalence of psoriasis and heel enthesitis was observed (33.3 and 72%, respectively). Other characteristics of the patients, including typical SpA features, have been reported in Table 1 and have previously been published (19). Out of all 75 patients, 37.3, 56, 100, and 64%, respectively, complained of cervical/thoracic/lumbar/buttock pain. We found a higher prevalence of HLA-B27, male gender, uveitis, increased serological markers and alternating buttock pain among subjects with MRI-SIJ+ for BMO lesions; instead a higher prevalence of inflammatory bowel diseases (IBD) in those with MRI-spine+ for BMO lesions. The other SpA features were comparable between subjects with MRI-SIJ+ and/or MRI-spine+ (Table 1). The clinical (MASES, BASMI), disease activity (BASDAI, ASDAS), functional (HAQ, BASFI, VAS pain, VAS pain night, VAS disease activity, BASG1, BASG2) indices as well as inflammatory biomarkers (ESR, CRP) were analyzed and measured at T0, T6, T12, and T24. Out of all 75 patients, 90.7% were evaluated at **T6**, of these 78.7% patients at **T12**, of these 72% at **T24**.

**Table 1:** Baseline features of all LBP patients in relation to the presence of BME lesions on MRI-spine and on MRI-SIJ.

**Baseline features of all patients with inflammatory LBP**	**Total patients *n* = 75**	**MRI-SIJ + / MRI-SPINE ±, *n* = 48**	**vs**.	**MRI-SIJ – / MRI-SPINE ±, *n* = 27**	**p§**	**MRI-SPINE + / MRI-SIJ ±, *n* = 44**	**vs**.	**MRI-SPINE – / MRI-SIJ ±, *n* = 31**	**p§**
Age of onset LBP, mean (± SD)	28.51 (± 8.05)	28.44 (± 7.24)		28.63 (± 9.48)	ns	28.11 (± 7.92)		29.06 (± 8.33)	ns
Male, n (%)	34 (45.3%)	27 (56.25%)		7 (25.93%)	<0.05	20 (45.45%)		14 (45.16%)	ns
Duration (months) di LBP, mean (± SD)	13.37 (± 6.14)	14.25 (± 6.38)		11.81 (± 5.47)	ns	13.77 (±.67)		12.81 (± 5.39)	ns
Only axial involvement, n (%)	39 (52%)	25 (52.08%)		14 (51.85%)	ns	25 (56.82%)		14 (45.16%)	ns
Axial and peripheral involvment, n (%)	36 (48%)	23 (47.92%)		13 (48.15%)	ns	19 (43.18%)		17 (54.84%)	ns
HLA-B27 positive, n (%)	29 (38.7%)	26 (54.17%)		3 (11.11%)	<0.05	19 (43.18%)		10 (32.26%)	<0.05
Positive family history of SpA, n (%)	35 (46.7%)	24 (50%)		11 (40.74%)	ns	20 (45.45%)		15 (48.39%)	ns
Peripheral arthritis, n (%)	34 (45.3%)	23 (47.92%)		11 (40.74%)	ns	19 (43.18%)		15 (48.39%)	ns
Psoriasis, n (%)	25 (33.3%)	17 (35.42%)		8 (29.63%)	ns	13 (29.55%)		12 (38.71%)	ns
Dactylitis, n (%)	15 (20%)	8 (16.67%)		7 (25.93%)	ns	5 (11.36%)		10 (32.26%)	ns
Heel enthesitis, n (%)	54 (72%)	33 (68.75%)		21 (77.78%)	ns	29 (65.91%)		25 (80.65%)	ns
Uveitis, n (%)	7 (9.3%)	7 (14.58%)		0 (%)	<0.05	4 (9.09%)		3 (9.68%)	<0.05
IBD, n (%)	9 (12%)	4 (8.33%)		5 (18.52%)	<0.05	7 (15.91%)		2 (6.45%)	<0.05
Preceding infections, n (%)^†^	4 (5.3%)	3 (6.25%)		1 (3.70%)	ns	2 (4.55%)		2 (6.45%)	ns
Good response to NSAIDs, n (%)	73 (97.3%)	47 (97.92%)		26 (96.30%)	ns	43 (97.73%)		30 (96.77%)	ns
Elevated CRP/ESR, n (%)	42 (56%)	29 (60.42%)		13 (48.15%)	<0.05	24 (54.55%)		18 (58.06%)	ns
Cervical pain, n (%)	28 (37.3%)	16 (33.33%)		12 (44.44%)	ns	18 (40.91%)		10 (32.26%)	ns
Thoracic pain, n(%)	42 (56%)	31 (64.58%)		11 (40.74%)	ns	25 (56.82%)		17 (54.84%)	ns
Buttock pain, n (%)	48 (64%)	35 (72.92%)		13 (48.15%)	<0.05	31 (70.45%)		17 (54.84%)	ns
Alternating buttock pain, n (%)	37 (49.3%)	29 (60.42%)		8 (29.63%)	<0.05	26 (59.10%)		11 (35.48%)	<0.05
Morning stiffness, n (%)	57 (76%)	37 (77.08%)		20 (74.07%)	ns	36 (81.82%)		21 (67.74%)	ns
Night pain, n(%)	71 (94.7%)	46 (95.83%)		25 (92.60%)	ns	43 (97.73%)		28 (90.32%)	ns
Sacroiliitis MRI ^*^, n (%)	48 (64%)	48 (100%)		0 (0%)	<0.05	29 (65.91%)		19 (61.29%)	<0.05
Sacroiliitis x-ray ^**^, n (%)	25 (33.3%)	19 (39.58%)		6 (22.22%)	<0.05	17 (38.64%)		8 (25.81%)	<0.05
Weight (kg), mean (± SD)	70.22 (± 16.15)	71.25 (± 15.87)		68.05 (± 16.98)	ns	66.60 (± 11.12)		75.5 (± 20.65)	ns
Height (cm), mean (± SD)	170.6 (± 8.67)	172.23 (± 8.73)		167.71 (± 7.91)	ns	170.41 (± 8.42)		170.88 (± 9.15)	ns

*HLA-B27, human leukocyte antigen; LBP, low back pain; IBD, inflammatory bowel disease; CRP, C-reactive protein; ESR, erythrocyte sedimentation rate; MRI, magnetic resonance imaging; SpA, spondyloarthritis; SIJ, sacroiliac joints; NSAID, non-steroidal antiinflammatory drugs; MRI+, presence of inflammatory lesions; MRI–r, absence of inflammatory lesions; ±, presence or absence of inflammatory lesions*.

* *sacroiliitis on MRI according ASAS/EULAR criteria*.

** sacroiliitis on X-Rays according modified New York criteria (0–4).

† *balanitis, urethritis, or cervicitis. p§, anova (Kruskal Wallis) a t0: p < 0.05; SD = deviation standard*.

### (A) The Prevalence of the Inflammatory and Structural Lesions on MRI at T0

The inter-observer reliability of MRI-SIJ and MRI-spine between two expert readers was good to moderate (kappa 0.75 for inflammatory lesions and 0.62 for structural lesions on MRI-spine; kappa 0.76 for inflammatory lesions and 0.64 for structural lesions on MRI-SIJ, respectively). The inter-observer reliability of X-rays was good (kappa 0.81 for spine radiological lesions and kappa 0.79 for SIJ radiological lesions). Moreover, intra-observer reliability was good for all spine and SIJ images on X-rays and MRI (respectively, kappa 0.78 for spine and 0.84 for SIJ on X-Rays and kappa 0.80 for spine and 0.82 for SIJ on MRI). Fifty-two (69.3%) patients presented structural and/or inflammatory lesions on MRI-SIJ at T0 (on the right SIJ in 63% of the patients and on left one in 59% of the patients). BMO lesions were observed in 48 (64%) patients (58% on the right SIJ and 50% on the left one) and structural lesions on MRI-SIJ in 33 (44%) patients (37% on the right SIJ and 30% on the left one). Fifty (66.7%) patients had inflammatory and/or structural lesions on the MRI-spine at T0. BMO lesions on the anterior corner of the spine were observed in 42 (56%) patients (cervical/thoracic/lumbar regions: 19, 39, and 33%, respectively). Structural spine lesions were detected in 28 (37.3%) patients (cervical/thoracic/lumbar regions: 18, 14, and 19%, respectively). Signs of enthesitis were found in 47 (62.7%) patients: (cervical/thoracic/lumbar spine: 8, 58, and 11%, respectively). At T0 18 patients (8 fulfilling *axSpA clinical*±*imaging arm* and 10 not fulfilling ASAS axSpA criteria) with inflammatory lesions on MRI-spine showed no abnormalities in SIJ, while 11 (14.7%) patients without active sacroiliitis on MRI-SIJ did not present MRI-spine lesions.

### (B) The Prevalence and Type of MRI Lesions in the 3 Cohorts at T0 and T24

The prevalence of inflammatory and structural MRI lesions in the three cohorts is outlined in Table 2. As it could be expected, an increased prevalence of structural lesions on MRI-SIJ was found at T0 in the *axSpA imaging arm* and *axSpA clinical*±*imaging arm* compared to those who did *not fulfill ASAS axSpA criteria*. In the cohorts who met axial ASAS criteria, a higher prevalence of inflammatory and structural spinal lesions was observed, as well as a decrease of MRI lesions during the follow-up period.

**Table 2:** The prevalence of inflammatory and structural lesions at T0 and T24 in three cohorts (*axSpA imaging arm, axSpA clinical* ± *imaging arm, not full ASAS axSpA*).

	**T0 MRI evaluation**		
	**axSpA imaging arm**	**axSpA clinical ± imaging arm**	**Not full ASAS axSpA**
Total number of patients	21 (28%)	29 (38.7%)	25 (33.3%)
SIJ total lesions	21 (100%)	27 (93.1%)	4 (16%)
BMO lesions	21 (100%)	27 (93.1%)	0 (0%)
Sclerosis lesions	10 (47.6%)	14 (48.28%)	0 (0%)
Fatty lesions	7 (33.3%)	2 (6.9%)	4 (16%)
Erosions lesions	8 (38.1%)	3 (10.3%)	0 (0%)
Spine total lesions	20 (95.2%)	19 (65.5%)	11 (44%)
BMO lesions	18 (85.7%)	15 (51.7%)	9 (36%)
Enthesitis lesions	17 (80.9%)	19 (65.5%)	11 (44%)
Fatty lesions	6 (28.6%)	7 (24.1%)	4 (16%)
Sclerosis/syndesmophytes lesions	6 (28.6%)	6 (20.7%)	3 (12%)
Erosions lesions	2 (9.5%)	2 (6.9%)	1 (4%)
	**T24 MRI evaluation**		
	**axSpA imaging arm**	**axSpA clinical ± imaging arm**	**Not full ASAS axSpA**
Total number of patients	16 (29.6%)	22 (40.7%)	16 (29.6%)
SIJ total lesions	16 (100%)	15 (68.2%)	1 (6.3%)
BMO lesions	9 (56.3%)	13 (59.1%)	0 (0%)
Sclerosis lesions	7 (43.8%)	10 (45.5%)	0 (0%)
Fatty lesions	6 (37.5%)	4 (18.2%)	1 (6.3%)
Erosions lesions	3 (18.8%)	2 (9.1%)	0 (0%)
Spine total lesions	11 (68.8%)	4 (18.2%)	7 (43.8%)
BMO lesions	7 (43.8%)	5 (22.7%)	5 (31.3%)
Enthesitis lesions	7 (43.8%)	6 (27.3%)	7 (43.8%)
Fatty lesions	6 (37.5%)	4 (18.2%)	4 (25%)
Sclerosis/syndesmophytes lesions	7 (43.8%)	3 (13.6%)	2 (12.5%)
Erosions lesions	1 (6.3%)	2 (9.1%)	0 (0%)

*MRI, magnetic resonance imaging; SIJ, sacroiliac joints; BMO, bone marrow oedema; axSpA, Axial Spondyloarthritis; T0, 0 months; T24, 24 months*.

### (C) Analysis of Clinical, Serological, Disease Activity, Imaging Indices in Overall Patients, and in the 3 Cohorts Over 24 Months Follow-Up

**Figure 3:**
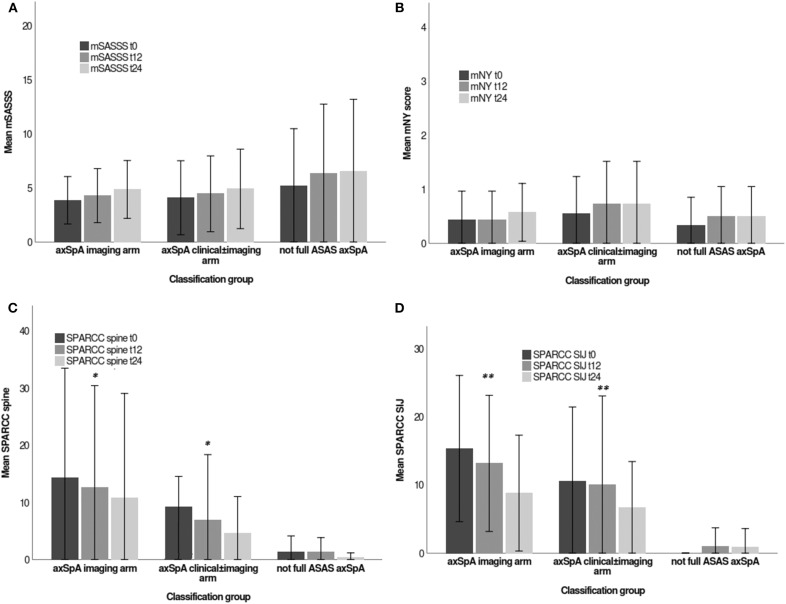
**(A–D)** Imaging indices values (mSASSS, mNY score, SPARCC spine, SPARCC SIJ), expressed as mean (deviation standard) from T0 to T24 in the three cohorts (*axSpA imaging arm, axSpA clinical*±*imaging arm, not full ASAS axSpA*). *p < 0.01 vs. T0, **p < 0.001 vs. T0. Stoke Ankylosing Spondylitis Spinal Score (SASSS) system modified by Creemers (mSASSS); modified criteria of New York (mNY) score; Spondyloarthritis Research Consortium of Canada (SPARCC); Sacroiliac joint (SIJ). The written informed consent was obtained from the individual for the publication of these images.

All indices (values expressed as mean and standard deviation-SD), analyzed by Kruskal-Wallis test are reported in Table 3 [data previously published elsewhere (19)]. Considering the whole population, a significant decrease in the following variables was noted from T0 to T24: MASES (*p* = 0.008), BASG1 (*p* = 0.02), BASG2 (*p* < 0.0001), HAQ (*p* = 0.0002), VAS pain (*p* = 0.01), VAS pain night (*p* = 0.04), VAS disease activity (*p* = 0.05), BASFI (*p* = 0.02), BASDAI (*p* < 0.0001), ASDAS (*p* < 0.0001). Conversely, BASMI, ESR, and CRP did not significantly decrease. Considering the patients subdivided in the 3 cohorts, a downward trend in all functional and disease activity indices was observed, which in some cases was significant (see Table 3); however, no significant differences were found among the cohorts. A significant downtrend of SPARCC SIJ and SPARCC spine score was also observed in *axSpA imaging arm* and *axSpA clinical*±*imaging arm* cohorts (Figures 3A–D).

**Table 3:** Clinical, functional, disease activity, and serological indices values from T0 to T24 in the whole group of patients (*n* = 75) and in three cohorts (axSpA imaging arm, axSpA clinical ± imaging arm, not full ASAS axSpA).

		**BASMI**	**MASES**	**BASFI**	**HAQ**	**BASG1**	**BASG2**	**VAS pain**	**VAS dis act**	**VAS pain N**	**BASDAI**	**ASDAS**	**ESR**	**CRP**
axSpA imaging arm	T0	1 (1.18)	3.33 (2.65)	17.43 (20.21)	0.34 (0.53)	3.43 (2.79)	5.71 (3.02)	3.48 (2.82)	3.81 (3.03)	3.24 (3.33)	39.50 (25.99)	2.29 (0.86)	17.52 (12.98)	4.81 (3.61)
	T6	0.80 (1.15)	3.30 (2.85)	17.95 (22.52)	0.21 (036)	3.15 (2.87)	4.80 (2.48)	3.40 (2.72)	3.35(2.91)	2.90 (2.92)	29.17 (26.19)	2.14 (0.98)	13.55(11.59)	3.04 (2.01)
	T12	0.39 (0.78)	3.06 (2.98)	12.67 (12.82)	0.15 (0.31)	2.01(2.03)	3.71 (2.44)	3.47 (2.61)	3.18 (2.74)	2.06 (2.34)	29.06 (26.71)^*^	1.78 (0.87)^*^	15.06 (10.23)	4.06 (2.98)
	T24	0.44 (0.63)	2.06 (2.11)^*^	9.25 (8.12)^**^	0.14 (0.38)^**^	2.29 (2.23)^*^	2.71 (2.64)^**^	1.75 (1.77)^*^	1.81 (1.71)^*^	1.56 (1.93)^*^	18.72 (18.25)^**^	1.36 (0.56)^**^	11.13(5.73)^**^	3.13 (1.31)
axSpA clinical ± imaging arm	T0	0.76 (1.02)	3.41 (2.35)	13.680 (15.14)	0.30 (0.38)	3.52 (2.77)	5.10 (2.77)	4.10 (2.97)	4.07 (3.15)	4.17 (3,57)	44.27 (25.03)	2.61 (0.56)	15.14 (11.76)	3.17 (3.32)
	T6	0.54 (0.86)	2.58 (2.39)	15.85 (20.65)	0.21 (0.37)	2.73(2.54)	3.84 (2.31)	3.08 (2.64)	3.19 (2.80)	2.62 (2.70)	35.11 (24.70)	1.95 (0.79)	14.88 (9.27)	3.12 (2.19)
	T12	0.43 (0.79)	2.39 (2.90)	11.04 (15.63)	0.10 (0.25)	2.67 (2.33)	3.33 (2.12)	2.41 (2.04)^*^	2.92 (2.22)	2.25 (2.26)^*^	25.48 (17.78)^**^	1.59 (0.54)^*^	12.73 (9.39)	4.05 (6.31)
	T24	0.50 (0.86)	2.09 (2.37)^*^	10.27 (14.29)^*^	0.10 (0.23)^*^	1.50 (1.87)^**^	1.91 (1.98)^**^	2.23 (2.20)^*^	2.18 (2.32)^*^	2.36 (2.32)^*^	20.73 (18.76)^***^	1.33 (0.69)^***^	10.86 (5.54)^*^	3.59 (3.84)
Not full ASAS axSpA	T0	0.92 (1.08)	3.56 (2.47)	22.44 (25.75)	0.52 (0.55)	4.64 (3.35)	5.28 (2.94)	5.08 (3.23)	5.04 (3.35)	4.20 (3.33)	53.48 (24.97)	2.66 (0.88)	21.68 (21.19)	4.24 (3.28)
	T6	0.96 (1.22)	3.43 (2.59)	17.52 (21.71)	0.34 (0.41)	4.26 (3.01)	4.82 (3.52)	4.52 (3.36)	4.30 (3.21)	3.48 (3.17)	45.66 (27.37)	2.51 (1.19)	19.73 (17.01)	6.35 (10.33)
	T12	0.65 (0.81)	2.40 (2.14)	12.38 (14.63)^*^	0.20 (0.29)	3.35 (2.69)	3.18 (2.46)	3.58 (3.17)	4.70 (3.16)	3.10 (3.01)	31.26 (19.67)^*^	2.02 (0.99)	16.06 (13.06)	4.22 (3.67)
	T24	0.50 (0.73)	2.40 (2.14)^*^	12.84 (12.63)^**^	0.15 (0.22)^**^	3.33 (2.19)^*^	3.67 (2,32)^*^	2.81 (2.32)^**^	2.81 (2.26)^*^	3.00 (2.59)^*^	24.80 (19.67)^**^	1.34 (0.61)^*^	14.25 (10.21)^**^	3.63 (2.03)
Total patients with IBP, *n* = 75	T0	0.88 (1.08)	3.44 (2.45)	17.65 (20.64)	0.39 (0.49)	3.87 (2.99)	5.33 (2.87)	4.25 (3.05)	4.32 (2.18)	3.92 (3.40)	46.01 (25.07)	2.54 (0.77)	17.99 (15.85)	3.98 (3.42)
	T6	0.75 (1.08)	3.07 (2.59)	17.01 (21.26)	0,25 (0,38)	3.36 (2.83)	4.45 (2.82)	3.65 (2.95)	3.61 (2.97)	2.98 (2.91)	36.90 (26.51)	2.19 (1.01)	16.09 (13.05)	4.29 (6.33)
	T12	0.49 (0.79)	2.59 (2.67)	11.95 (14.30)^*^	0,16 (0,30)^**^	2.67 (2.38) ^*^	3.40 (2.29)^**^	3.08 (2.61)	3.57 (2.77)	2.47 (2.54)	28.37 (21.05)^**^	1.79 (0.82)^**^	14.48 (10.80)^*^	4.10 (4.62)
	T24	0.48 (0.75)	2.12 (2.13)^**^	10.73 (12.12)^**^	0,14 (0,29)^***^	2.25 (2.17) ^**^	2.63 (2.24)^***^	2.26 (2.12)^**^	2.26 (2.14)^**^	2.28 (2.30)^**^	21.34 (18.07)^***^	1.34 (0.61)^***^	11.94 (7.30)^**^	3.46 (2.75)

*Data are expressed as mean ± SD. The test Kruskal Wallis repeated measures test and Dunn's multiple comparison test were used*.

*** p < 0.0001 vs. T0,

** p < 0.001 vs. T0,

* * < 0.01 vs. T0*.

*BASMI, bath ankylosing score metrology index; MASES, maastricht ankylosing spondilities enthesitis score; BASFI, bath ankylosing spondylitis functional index; HAQ, health assessment questionnaire; BASG1, bath ankylosing spondylitis patient global score 1; BASG2, bath ankylosing spondylitis patient global score 2; ESR, erytrocyte sedimentation rate; CRP, C-reactive protein; BASDAI, bath ankylosing spondylitis disease activity index; ASDAS, ankylosing spondylitis disease activity score; VAS pain, visual analog scale pain; VAS dis act, visual analog scale disease activity; VAS pain N, visual analog scale pain night*.

### (D) Regression Analysis to Identify the Predictors of Disease Activity and Radiological Progression

A regression analysis was performed to identify any baseline predictors of radiological activity on MRI at T24 (measured by continuous variables such as SPARCC SIJ and SPARCC spine) and of structural damage at T24 (measured by continuous variables such as mSASSS and SIJ mNY score and dichotomous variables such as presence/absence of structural lesions on X rays of SIJ and spine). As shown in Tables 4–6, at the multivariate analysis radiological spine structural damage was independently predicted by an early onset of LBP, a lower use of NSAIDs, a BASDAI>4. Radiological SIJ structural damage was independently predicted by high SPARCC SIJ score. A higher mSASSS was independently predicted by a lower age of onset of LBP. Predictive factors of increased radiological activity were: a higher NSAIDs intake for a higher SPARCC spine score and the HLA-B27 positivity and increased serological inflammatory markers for a higher SPARCC SIJ score, respectively.

**Table 4:** Baseline predictors factors of mSASSS score at T24.

**Independent variables**	**Univariable analysis**		**Multivariable analysis**	
	**coeff (IC)**	***p***	**coeff (IC)**	***p***
Female sex	−0.80 (−2.55, 0.93)	0.359	–	–
LBP onset age	0.21 (0.11, 0.30)	0.000	0.19 (0.09, 0.29)	0.000
Duration of LBP	0.02 (−0.12, 0.16)	0.770	–	–
HLA-B27+	−0.72 (−2.5, 1.08)	0.428	–	–
Increased ESR/CRP	0.97 (−0.80, 2.75)	0.278	–	–
BASDAI > = 4	−0.48 (−2.22, 1.26)	0.583	–	–
Use of NSAIDs	2.63 (−0.22, 5.48)	0.070	1.22 (−1.46, 3.90)	0.367
SPARCC SIJ >2	0.91 (−0.84, 2.55)	0.233	–	–

*LBP, low back pain; HLA-B27, human leukocyte antigen B27; CRP, C-reactive protein; ESR, erythrocyte sedimentation rate; BASDAI, bath ankylosing spondylitis disease activity index; NSAIDs, non-steroidal antiinflammatory drugs; SPARCC, spondyloarthritis research consortium of Canada; SIJ, sacroiliac joints; mSASSS, modified Stoke ankylosing spondylitis spinal score (SASSS) system; T24, 24 months*.

**Table 5:** Baseline predictors factors of SPARCC spine score and SPARCC SSJ score at T24.

**SPARCC spine score**				
**Independent variables**	**Univariable analysis**		**Multivariable analysis**	
	**coeff (IC)**	***p***	**coeff (IC)**	***p***
Female sex	−0.14 (−0.56, 0.28)	0.511	–	–
LBP onset age	−0.01 (−0.04, 0.00)	0.152	−0.02 (−0.05, 0.00)	0.124
Duration of LBP	0.02 (−0.01, 0.05)	0.154	0.02 (−0.01, 0.05)	0.192
HLA-B27+	0.35 (−0.06, 0.78)	0.099	0.12 (−0.33, 0.58)	0.582
Increased ESR/CRP	0.11 (−0.31, 0.54)	0.583	–	–
BASDAI>=4	–0.39(−0.81, 0.01)	0.058	–0.33 (–.73, 0.07)	0.104
Use of NSAIDs	0.60 (–0.26, 1.48)	*0*.168	0.88 (0.01, 1.75)	0.046
SPARCC SIJ>2	0.44 (−0.42, 0.65)	0.311	–	–
**SPARCC SSJ score**				
**Independent variables**	**Univariable analysis**		**Multivariable analysis**	
	**coeff (IC)**	***p***	**coeff (IC)**	***p***
Female sex	−3.59 (−9.54, 2.36)	0.233	–	–
LBP onset age	−0.36 (−0.72, –0.004)	0.047	−0.25 (−0.60, 0.08)	0.140
Duration of LBP	0.00 (−0.48, 0.49)	0.980	–	–
HLA-B27+	9.41 (3.64, 15.18)	*0*.002	8.87 (3.08, 14.65)	0.003
Increased ESR/CRP	4.46 (–1.59, 10.51)	*0*.146	5.74 (0.15, 11.34)	0.044
BASDAI > = 4	−3.94 (−9.85, 1.96)	0.187	−3.14 (−8.55, 2.26)	0.250
Use of NSAIDs	3.87 (−6.09, 13.84)	0.441	–	–

*LBP, low back pain; HLA-B27, human leukocyte antigen B27; CRP, C-reactive protein; ESR, erythrocyte sedimentation rate; BASDAI, bath ankylosing spondylitis disease activity index; NSAIDs, non-steroidal antiinflammatory drugs; SPARCC, spondyloarthritis research consortium of Canada; SIJ, sacroiliac joints; T24, 24 months*.

**Table 6:** Baseline predictors factors of spine or pelvis structural lesions at T24.

**Spine structural lesions (presence = 1; absence = 0)**				
**Independent variables**	**Univariable analysis**		**Multivariable analysis**	
	**OR (IC)**	***p***	**coeff (IC)**	***p***
Female sex	0.93 (0.69, 1.26)	0.661	–	–
LBP onset age	0.90 (0.84, 0.97)	0.006	0.89 (0.82, 0.97)	0.010
Duration of LBP	0.97 (0.90, 1.05)	0.495	–	–
HLA-B27+	1.22 (0.48, 3.08)	0.672	–	–
Increased ESR/CRP	0.46 (0.16, 1.31)	0.147	0.57 (0.16, 1.93)	0.369
BASDAI > = 4	2.15 (0.79, 5.85)	0.131	4.05 (1.15, 14.24)	0.029
Use of NSAIDs	0.07 (0.008, 0.65)	0.019	0.09 (0.008, 0.99)	0.049
SPARCC SIJ >2	1.28 (0.59, 2.44)	0.531	–	–
**Pelvis structural lesions (presence = 1; absence = 0)**				
**Independent variables**	**Univariable analysis**		**Multivariable analysis**	
	**OR (IC)**	***p***	**coeff (IC)**	***p***
Female sex	0.45 (0.17, 1.18)	0.107	0.65 (0.21, 2.01)	0.462
LBP onset age	0.98 (0.92, 1.03)	0.527	–	–
Duration of LBP	1.00 (0.92, 1.08)	0.951	–	–
HLA-B27+	1.45 (0.58, 3.60)	0.423	–	–
Increased ESR/CRP	1.27 (0.48, 3.35)	0.629	–	–
BASDAI > = 4	0.43 (0.16, 1.14)	0.091	0.60 (0.19, 1.84)	0.375
Use of NSAIDs	1.77 (0.32, 9.84)	0.511	–	–
SPARCC SIJ >2	1.07 (1.026, 1.13)	0.003	1.07 (1.01, 1.12)	0.007

*LBP, low back pain; HLA-B27, human leukocyte antigen B27; CRP, C-reactive protein; ESR, erythrocyte sedimentation rate; BASDAI, bath ankylosing spondylitis disease activity index; NSAIDs, non-steroidal antiinflammatory drugs; SPARCC, spondyloarthritis research consortium of Canada; SIJ, sacroiliac joints; T24, 24 months; OR, Odds Ratio*.

### (E) Evaluation of the Pelvic and Spinal Radiographic Progression From T0 to T24

In our study SIJ radiographic progression (17) and spine radiographic progression (18) were not significant from T0 to T24.

## Discussion

Our study highlighted the presence of BMO both in the SIJ and in the spine, especially the thoracic spine. BMO seemed to be positively associated to HLA-B27 and inflammatory serological indices at the SIJ level, moreover it was negatively associated with NSAIDs use at the spine level. When present, BMO tended to decrease during follow-up, after beginning an adequate therapy for axSpA.

**Figure 4:**
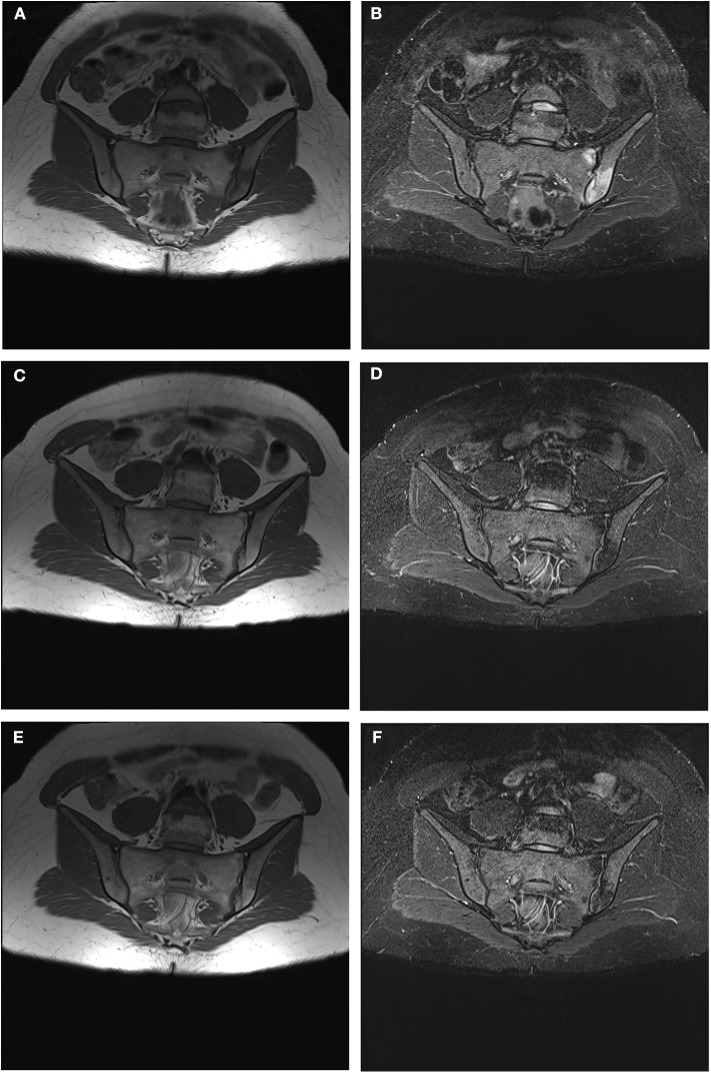
**(A–F)** Progressive reduction until disappearance of BMO signs and subsequent corresponding appearance of signs of adipose metaplasia on left SIJ during a follow up period of 24 months. **(A)** MRI-SIJ at T0 in T1 sequence **(B)** MRI-SIJ at T0 in STIR sequence **(C)** MRI-SIJ at T12 in T1 sequence **(D)** MRI-SIJ at T12 in STIR sequence **(E)** MRI-SIJ at T24 in T1 sequence **(F)** MRI-SIJ at T24 in STIR sequence. The written informed consent was obtained from the individual for the publication of these images.

The advent and development of new imaging methods in the last two decades has increased the interest to diagnose axSpA forms in the earlier stages—before structural damage has occurred—aiming at an earlier and more effective treatment (3, 4, 20, 21). MRI has a high sensitivity in detection of inflammatory lesions of the spine and SIJ, hence its widespread use in clinical practice, especially in assessing patients with suspected axSpA and/or a history of chronic inflammatory LBP (6, 7, 22–25). MRI-SIJ is useful since it reveals the pathological lesions which led to an axSpA diagnosis (6). Based on the definition of the ASAS/OMERACT criteria, an MRI-SIJ is considered positive if at least one BMO, highly suggestive for SpA, is present on ≥2 consecutive slices or if ≥2 bone inflammatory lesions are visible on a single slice (26, 27). Nevertheless, a SPARCC score >2—widely used because it measures inflammation on a continuous scale with good sensitivity to change (15, 16)—can be used to define a positive MRI-SIJ (ASAS definition) in clinical trials (28). Some studies have recently considered inflammatory and structural lesions observed on MRI-spine in SpA patients (7, 22): the most frequent and specific lesions are the BMO of the anterior vertebral corners, which is an expression of anterior osteitis (29); other possible lesions are fatty lesions—replacement of vertebral corners with fatty tissue—though they appear to be less specific for SpA and of later appearance (30–32). Likewise to previous studies, including those from ASAS/OMERACT MRI study group, the prevalence and type of both inflammatory and structural lesions in our study were analyzed in subjects with LBP and suspected early axSpA. In our study population, a higher prevalence of BMO lesions compared to structural lesions, has been noted at MRI, confirming the ability of this method in the visualization of inflammatory lesions. A significant prevalence of BMO lesions was observed in both SIJs and the spinal district, with predominant localization in the vertebral anterior corner. This observation, in keeping with previous studies, highlights the importance of spine involvement in the initial stage of the inflammatory process in axSpA (3, 29, 33, 34). This data are especially interesting in the nr-axSpA forms without sacroiliitis on MRI, suggesting that the inclusion of spine BMO lesions at MRI among the classification criteria for axSpA would decrease the number of false negative patients. In some studies, it has been shown that the determination of ≥3 inflammatory lesions (BMO) in the anterior vertebral site (anterior spondylitis) in subjects younger than 45 years increases the likelihood of axSpA diagnosis (32, 35, 36). On the other hand, although the appearance of multiple structural (at least three) and *fatty* lesions increases the probability of axSpA (37), it has been reported that these lesions tend to increase with aging and can be found even in healthy individuals or in those affected with other spinal degenerative diseases (38, 39). In a recent study the presence on MRI of ≥ 5 spinal inflammatory lesions or ≥ 5 spinal fatty lesions—unlike the presence of ≥3 spinal lesions—appears to discern between axSpA patients and non-SpA patients, while maintaining >95% specificity (40). Furthermore, while an association between inflammatory MRI lesions and LBP has been confirmed, it remains unclear whether axSpA spinal lesions could be associated with pain (41). Therefore, the inclusion of MRI-spine lesions in classification criteria is currently under discussion owing to its increased sensitivity—albeit with a reduced specificity. In the present study, MRI-spine also showed a high prevalence of inflammatory lesions attributable to enthesitis (47 patients) mostly in the thoracic district (58%) (42), suggesting an early involvement of this site in the initial stage of axSpA. We found no significant variations in the radiological progression of SIJ and spine evaluated by SIJ mNY and mSASSS scores from T0 to T24, according to definitions used in previous studies (17, 18). These results may be explained by the very early disease stage of our patients and the relatively short follow-up (24 months is a minimal timeframe to observe any significant radiological changes). A downward trend of radiological activity measured by ΔSPARCC SIJ and ΔSPARCC spine and of prevalence of inflammatory lesions on spinal and pelvic MRIs was observed in the 2 cohorts that met the ASAS criteria, probably due to the pharmacological treatment initiated after baseline evaluation and the diagnosis of axSpA (Figures 4A–F). However, due to the non-homogeneity in therapy and reduced sample size (*n* = 54 patients) at T24 with available imaging, a comparative analysis on the ΔSPARCC SIJ and the ΔSPARCC spine by treatment type was not possible. Several studies systematically evaluated the concomitant use of MRI-spine and MRI-SIJ in patients with suspected axSpA and healthy controls, highlighting an improved diagnostic capacity for the two techniques together compared to MRI-SIJ only (8, 43). However, other authors claim that this procedure only increases the level of diagnostic probability in patients with suspected axSpA due to the inclusion of false positives (37–39, 44). In another recent study the prevalence of lesions on MRI-spine in patients with ≤3-year duration of chronic LBP included in SPACE and DESIR cohorts, was found in very few patients without sacroiliitis on X-rays or MRI-SIJ: 3/447 (1%) patients in SPACE cohort and 8/382 (2%) in DESIR cohort, respectively. The conclusion of this study was that the addition of MRI-spine in ASAS axSpA criteria yielded few newly classified patients (45). On the opposite, our study suggests that the use of MRI-spine along with MRI-SIJ could add a relevant information which can be useful in the diagnosis and in the the follow-up. In fact, at T0 we found 24% patients with positive MRI-spine and negative MRI-SIJ, as well as 14.7% patients with negative MRI-spine and positive MRI-sacroiliitis. Predictors of radiological damage and activity in our population were: early age of disease onset and long duration of LBP, increased inflammatory biomarkers, higher use of NSAIDs, male gender, HLA-B27 positivity and a SPARCC SIJ score>2, in keeping with previous studies (46, 47). Moreover, in our study we observed as curiuos aspect that patients with positive MRI-spine had higher prevalence of IBD, instead patients with positive MRI-SIJ presented more frequently uveitis. Our study also investigated whether there were differences in clinical indices of disease activity according to the presence or absence of signs of sacroiliitis on X-rays and MRIs. Despite the differences at T0 in the three cohorts in the prevalence of sacroiliitis on MRIs, X-rays, and SPARCC SIJ score, no differences were found in clinical and disease activity indices. Patients with active MRI sacroiliitis did not show higher disease activity indices compared to those without inflammatory changes in the SIJs or those with initial signs of radiographic sacroiliitis. In contrast, several studies reported higher values of clinical, functional and disease activity indices in AS and r-axSpA in patients with long disease duration vs. subjects with nr-axSpA (46, 48–52). In our study a significant reduction of functional and disease activity indices was observed in all patients throughout the follow-up period. Nevertheless, no markedly significant decrease of these indices was observed in one cohort vs. the others. The improvement of these indices may depend on the successful pharmacological treatment after the diagnosis of axSpA.

Some of the limitations of this study pertain to the small sample size, the early disease stage which could limit the potential to detect radiographic changes, and the variability of treatments in the three cohorts. Conversely, the strengths are the prospective study design and the central reading by two expert radiologists.

## Conclusions

A high prevalence of inflammatory lesions on MRI-SIJ and MRI-spine was found in our patients. Since inflammatory lesions on MRI-spine can occur in the absence of SIJ involvement, the use of MRI-spine alongside MRI-SIJ may add a relevant information which can be useful in the diagnosis, especially of nr-axSpA. As of today, no studies assessed the involvement of the thoracic spine by means of a spinal structural damage scoring method. Notably, we observed a significant involvement of the thoracic region in our patients which warrants further investigation. In the current study, different types of axial involvement—presence/absence of sacroiliitis—were not associated to significant differences in clinical severity and disease activity indices in all three cohorts.

## Data Availability Statement

The datasets generated for this study are available on request to the corresponding author.

## Ethics Statement

The studies involving human participants were reviewed and approved by the Medical Ethics Committee, Leiden University Medical Center (approval no. P08.105); and Azienda Ospedaliera di Padova (approval no. 2438P). The patients/participants provided their written informed consent to participate in this study.

## Author Contributions

ML participated in drafting the manuscript as well as gathering, analyzing, and interpreting the data. RR and AD conceived and designed the study, participated in data processing and drafting the manuscript. CL, SV, and VS participated in performing spine and pelvis X-rays and MRIs and participated in reading the images. AO, MFe, PP, and MFa participated in gathering, analyzing, and interpreting the data. All the authors have made substantive intellectual contributions to the study, have reviewed the article, and have given final approval for the version being submitted.

## Conflict of Interest

The authors declare that the research was conducted in the absence of any commercial or financial relationships that could be construed as a potential conflict of interest.
